# Helmet Ventilation for Pediatric Patients During the COVID-19 Pandemic: A Narrative Review

**DOI:** 10.3389/fped.2022.839476

**Published:** 2022-02-02

**Authors:** Shu-Chi Mu, Yu-Hsuan Chien, Pin-Zhen Lai, Ke-Yun Chao

**Affiliations:** ^1^Department of Pediatrics, Shin Kong Wu Ho-Su Memorial Hospital, Taipei, Taiwan; ^2^School of Medicine, College of Medicine, Fu Jen Catholic University, New Taipei City, Taiwan; ^3^Department of Pediatrics, Fu Jen Catholic University Hospital, Fu Jen Catholic University, New Taipei City, Taiwan; ^4^Department of Respiratory Therapy, Fu Jen Catholic University Hospital, Fu Jen Catholic University, New Taipei City, Taiwan; ^5^School of Physical Therapy, Graduate Institute of Rehabilitation Sciences, Chang Gung University, Taoyuan, Taiwan

**Keywords:** helmet ventilation, pediatric, non-invasive respiratory support, continuous positive airway pressure, non-invasive positive pressure ventilation, coronavirus disease 2019, respiratory failure

## Abstract

The air dispersion of exhaled droplets from patients is currently considered a major route of coronavirus disease 2019 (COVID-19) transmission, the use of non-invasive ventilation (NIV) should be more cautiously employed during the COVID-19 pandemic. Recently, helmet ventilation has been identified as the optimal treatment for acute hypoxia respiratory failure caused by COVID-19 due to its ability to deliver NIV respiratory support with high tolerability, low air leakage, and improved seal integrity. In the present review, we provide an evidence-based overview of the use of helmet ventilation in children with respiratory failure.

## Introduction

Coronavirus disease 2019 (COVID-19), which is caused by severe acute respiratory syndrome coronavirus 2 (SARS-CoV-2) (1), has been the cause of worldwide morbidity and mortality (2). Adults with COVID-19 can rapidly develop severe respiratory distress or acute respiratory failure (1, 3, 4). However, the number of cases of children with COVID-19 has been relatively small; furthermore, the disease is generally less severe in children than in adults and may exhibit different symptoms (5–9). Only a few cases of children with COVID-19 have been reported in which non-invasive respiratory support or mechanical ventilation was required (6, 7, 9, 10). For cases in which conventional oxygen therapy did not relieve respiratory symptoms, treatment was escalated to noninvasive ventilation (NIV) as respiratory support, which was then escalated to invasive ventilation if necessary (1, 3, 4). NIV, which has been well-established, may prevent clinical deterioration, the need for intubation, and intensive care unit admission (11, 12). Therefore, when fitting an NIV device, health care workers are at risk of contracting SARS-CoV-2 through virus-bearing droplets or exposure to air exhaled within a distance of 0.5–1.0 m (13, 14); air dispersion of exhaled droplets from patients with COVID-19 is considered a major route of disease transmission. An appropriate NIV interface must, therefore, be selected to protect health care workers. Recently, NIV using a helmet has attracted considerable attention and has been identified as an appropriate choice for patients with COVID-19 because it reduces air leakage (15–18). As an interface for NIV, the helmet offers several advantages, including higher tolerability, reduced air leakage, and improved seal integrity (19, 20). This narrative review offers an evidence-based overview of the use of helmet ventilation in pediatric populations with respiratory failure during the COVID-19 pandemic.

## Literature Search

We searched PubMed for all articles in English from January 2000 to June 2021 that referred to children or infants treated with helmet ventilation using the following keywords: “helmet ventilation,” “helmet continuous pressure airway pressure,” “helmet non-invasive positive pressure ventilation,” “neonatal helmet,” and “pediatric helmet.” The full texts or abstracts of all identified publications were screened, and three randomized controlled trials and five non-randomized studies were selected for review.

### Helmet Interface

**Figure 1:**
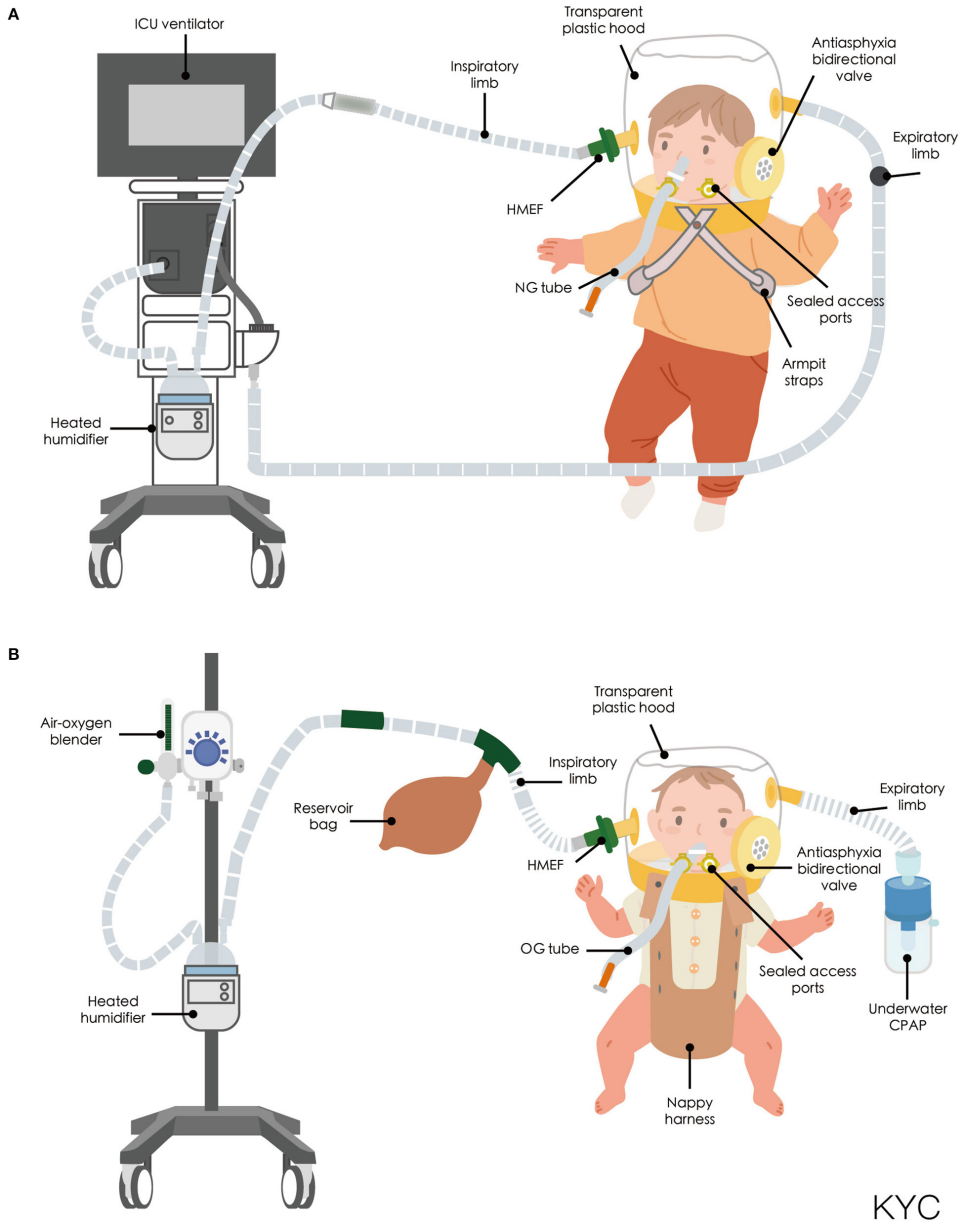
**(A)** Schematic of helmet connected to a ICU ventilator. **(B)** Schematic of helmet connected to a high-flow generator with an underwater positive end-expiratory pressure system. CPAP, continuous positive airway pressure; HMEF, Heat and moisture exchanging filter; ICU, intensive care unit; NG tube, nasogastric tube; OG tube, Orogastric tube.

Non-invasive positive pressure ventilation (NIPPV) and continuous positive airway pressure (CPAP) are usually administered via a facemask or nasal prong rather than a helmet. A large web-based European survey of 111 units from 23 different countries revealed that a helmet was more frequently available in Southern Europe (60.8%) than in Northern (11.8%) or Central Europe (23.4%) (21). CPAP was first described by Gregory et al. (22) and subsequently became the standard therapy for neonatal respiratory care. The first interface for neonatal CPAP is also known as a Gregory box, which is a forerunner of the helmet interface (22). The helmet used in NIV is a transparent, latex-free polyvinyl chloride hood, which was originally designed for hyperbaric oxygen therapy (23). Compared with the conventional NIV interface, the helmet surrounds the patient's head without coming into contact with the face and is sealed around the neck with a soft, latex-free polyurethane collar (24). The design of the helmet prevents complications that normally arise from the conventional interface, such as ulcerations of the nasal bridge and facial skin trauma (25–29). An antiasphyxia bidirectional valve is positioned on the outside of the head hood and can be manually opened to prevent suffocation if the source of the gas flow malfunctions or automatically activated when the pressure inside the helmet drops to 2–3 cmH_2_O. Basic nursing care can also be performed through the opening port (30). The helmet can be secured on the patient through two methods. For children and adult patients, the head hood and neck collar are connected by a hard plastic ring and are secured to the patient with padded armpit straps (Figure 1A), which are attached to hooks on the front and back of the hard plastic ring (24). For infants and small children, the helmet is secured using a baby-body device (30, 31), in which a “diaper” is worn under the pubic region that resembles a side-snap bodysuit (Figure 1B). CPAP of the helmet can be connected to a ventilator or a high-flow generator with either a positive end-expiratory pressure (PEEP) valve or an underwater PEEP system with a conventional breathing circuit. Sealed access ports on the neck collar can be used for nasogastric or orogastric tubes. To ensure the sealing effect is tight but comfortable, the size of the helmet is selected on the basis of the patient's body weight. The internal volume of the helmet hood for pediatric and neonatal patients is 7–8 liters. Because there is a potential risk of CO_2_ rebreathing due to the large internal volume (32–34), the continue gas flow must be delivered at 30–45 L/min for pediatric and neonatal patients (30, 35–37). However, depending on the ventilation device, CPAP bias flow cannot always be adjusted. To prevent patient–ventilator asynchrony caused by the large internal volume and minimize the required respiratory effort, the inspiratory rise time should be as short as possible, and the breathing cycling-off should be set at 50% of the peak inspiratory flow rate (38, 39).

### Noise Exposure

The turbulent flow inside the helmet creates a louder noise than conventional interfaces do for both adult (40, 41), and neonatal patients (42). High noise exposure has been proven to negatively affect infants by creating a potential risk for hearing impairment and irritation (43, 44). The American Academy of Pediatrics has recommended that environmental noise levels should not exceed 45 dBA for infants and warned that prolonged exposure to noise levels higher than 90 dBA may lead to hearing loss (45). For the CPAP of NIV helmets, the noise level is generally 76 and 117 dBA when the gas flow is set at 60 and 80 L/min, respectively; the noise level is affected by the gas source and flow rate. To reduce noise levels for CPAP in helmets, one study connected a heat and moisture exchange (HME) filter to the inspiratory limb closest to the helmet, which served as an exhaust gas muffler (46). However, another study reported that the use of HME did not reduce the noise level in a NIPPV helmet; rather, it decreased participants' perceptions of the noise level (41). In a neonatal population, a study indicated that the CPAP noise level inside the helmet was 42–78 dBA (47). Hernández-Molina et al. indicated that the use of HME attenuated noise levels at a flow rate of 20 L/min and increased noise levels at a flow rate of 40 L/min (48). However, data regarding perceived noise levels in infants are difficult to obtain. Although the use of HME as an exhaust gas muffler does not always affect the noise intensity measured by sound level meters, it decreases patients' perceived noise level. Furthermore, HME is inexpensive and easy to clinically implement.

### Humidification

Underhumidification can cause injury through inspiration of unhumidified gas; therefore, an active heated humidifier (HH) should be employed during helmet ventilation (49). Adequate humidification protects airway mucosa and can lead to fewer complications (50). Chiumello et al. investigated the humidity inside a helmet with high-flow generator CPAP and ventilator CPAP in adult participants (51). During ventilator CPAP without HH, the humidity levels inside the helmet were similar to those of the ambient air. However, the large internal volume and closed system of the helmet allowed the dry, inspired medical gas and the heated humidified gas expired by the patient to mix. Furthermore, during high-flow generator CPAP with and without HH, the humidity levels inside the helmet were lower than those during ventilator CPAP (51). Use of HH has been inconsistent in neonatal and pediatric populations. In an observational report of humidification status in helmet CPAP, the humidity inside the helmet was 98% when HH was turned on and decreased to 40% 1 h after HH was turned off (52). However, in adult populations, Codazzi et al. used an HH adjusted according to the tolerance and body temperature of the patients and reported no high degree of humidification (30). Chidini et al. did not use an HH during their short, 90-min trial (35); however, in their subsequent multicenter trial, an HH was intermittently used to provide airway humidity. To avoid over humidification of gases and rainout caused by the mixing chamber effect, nurses were asked to turn HHs off when rainout was observed inside the helmet (36). We suggest that the delivered gas flow be made to bypass an auto feeding water chamber and that HH be intermittently turned on as required.

### Helmet Ventilation in Children

Most clinical trials have used helmet CPAP rather than helmet NIPPV in small children with respiratory disorders (Table 1). Milési et al. demonstrated that a helmet was a feasible and well-tolerated interface for delivering CPAP to all infants, with condition stability and improvement observed in more than two-thirds of all infants (52). To investigate the efficacy of helmet CPAP in infants with acute respiratory failure (ARF), Chidini et al. conducted one single-center trial (35) and one multicenter trial (36). In the single-center, 90-min CPAP trial, 20 infants with ARF were enrolled; children receiving helmet CPAP had a lower treatment failure rate and a better tolerance for the treatment, which resulted in longer application time than that of facemask treatment. Although CPAP delivered through the facemask had a shorter application time, area redness over the contact points occurred more frequently than when CPAP was delivered using the helmet. Oxygenation levels increased in both interfaces; however, the transcutaneous CO_2_ tension levels were comparable. CPAP delivered through the helmet improved respiratory rates and respiratory effort scores compared with facemask delivery (35). In the multicenter trial, which enrolled a total of 30 infants with ARF, CPAP delivered through the helmet had a lower treatment failure rate. The main reason for treatment failure in infants using facemasks was intolerance to treatment; infants with intolerance were all successfully switched to helmet treatment. Therefore, the intubation rate was comparable between groups. Oxygenation levels increased in both groups; however, the PaCO_2_ levels were comparable. CPAP delivered through the helmet improved respiratory rates and comfort levels compared with facemask delivery (35). Both interfaces in the milticenter study had longer application times than that of the previous single-center study (35); the total application time of CPAP through the helmet was longer than that of the facemask (28 h vs. 8 h, *p* = 0.004). Although the application time was longer for the helmet group, air leaks and area redness over contact points were less frequent (35). Incidents of necessary sedation in infants were fewer in the CPAP helmet group compared with the CPAP facemask group in both studies (35, 36). Vitaliti et al. conducted a prospective observational study to evaluate the efficacy and safety of helmet CPAP with high-flow nasal cannula. Oxygenation improved in both the helmet CPAP and high-flow nasal cannula (HFNC) groups. PaCO_2_ and pH improved only in the helmet CPAP group. Respiratory rates were not affected in either group. However, helmet CPAP had a better clinical course compared with HFNC (56).

**Table 1:** Clinical trials of pediatric helmet CPAP and NIPPV.

**Reference**	**Type of study**	**Enrollment**	**Intervention**	**Results**
Piastra et al. (53)	Single-center, prospective observational case series	Children (9–17 y) with hypoxemic ARF	Helmet NIPPV, *n* = 4 PS: symptom relief^a^ PEEP: 5–12 cmH_2_O	Oxygenation improved above baseline. Helmet NIPPV was well-tolerated by all children. No major complications observed.
Codazzi et al. (30)	Single-center, prospective observational case series	Children (1 m−5 y) with hypoxemic ARF	Helmet CPAP, *n =* 15 CPAP: 5 or 10 cmH_2_O Flow rate: 30 L/min	Oxygenation improved above baseline. PaCO_2_ and respiratory rates remained comparable. Helmet was well-tolerated by all children. No major complications or mortality observed.
Mayordomo-Colunga et al. (54)	Single-center, prospective observational case series	Children (<3 m) with bronchiolitis	Helmet heliox-CPAP, *n =* 8 CPAP: 6–10 cmH_2_O Flow rate: 30 L/min	Helmet heliox-CPAP seemed feasible. No side effects or difficult-to-manage effects reported.
Piastra et al. (55)	Single-center, prospective case series	Immunocompromised children (1–18 y) with ARDS	Helmet NIPPV, *n =* 13 PS: initiated to achieve V_Te_ of 6 mL/kg and then increased for symptom relief^a^ PEEP: ≤ 12 cmH_2_O FiO_2_: set to maintain SaO_2_ ≥ 90%	Helmet NIPPV was well tolerated by all children. No major complications were observed.
Milési et al. (52)	Single-center, observational case series	Infants (1–12 m) with ARF	Helmet CPAP, *n =* 23 CPAP: 6 cmH_2_O Flow rate: >25 L/min FiO_2_: set to maintain SaO_2_ ≥ 90%	Fewer than one-third of the infants developed respiratory failure or deterioration. Only two infants required intubation due to severe laryngeal stridor. The setting of oxygen concentration was taper down, whereas SpO_2_ remained stable. PaCO_2_ and respiratory rates were similar to the baseline values.
Chidini et al. (35)	Single-center, prospective crossover RCT	Infants (1 m−2 y) with ARF	Helmet/facemask CPAP, *n =* 20 CPAP: 4–10 cmH_2_O Flow rate: >40 L/min	Helmet CPAP had a lower treatment failure rates and fewer infants requiring sedation. Oxygenation increased in both interfaces. PtcCO_2_ remained comparable. No major complications due to the interfaces reported. Facemask CPAP had higher rates of cutaneous sores and air leaks.
Chidini et al. (36)	Multicenter, prospective RCT	Infants (6 m−1 y) with RSV-induced ARF	Helmet CPAP, *n =* 15 CPAP: 4–10 cmH_2_O Flow rate: >35 L/min Facemask CPAP, *n =* 13 CPAP: 7.5 cmH_2_O Flow rate: >35 L/min	Helmet CPAP had lower treatment failure rates due to higher tolerance and fewer infants requiring sedation. Oxygenation increased in both interfaces. PaCO_2_ remained comparable. The intubation rate was similar in both groups. No major complications reported due to the interfaces. Facemask CPAP had higher rates of cutaneous sores and air leaks.
Vitaliti et al. (56)	Multicenter, prospective RCT	Infants (1–24 m) with respiratory distress	Helmet CPAP, *n =* 20 CPAP: 4–7 cmH_2_O Flow rate: 30 L/min HFNC, *n =* 20 Flow rate: 1–3 L/kg/min	Oxygenation improved quickly above baseline in both helmet and HFNC groups. Helmet CPAP had observed improvement in PaCO_2_ and pH values. Respiratory rates were similar both groups. Helmet CPAP had a better clinical course than HFNC CPAP.

a *Symptom relief: respiratory rate decrease and disappearance of accessory muscle activity. CPAP, continuous positive airway pressure; NIPPV, noninvasive positive pressure ventilation; ARF, acute respiratory failure; PS, pressure support; PEEP, positive end-expiratory pressure; PaCO_2_, arterial carbon dioxide tension; ARDS, acute respiratory distress syndrome; V_Te_, exhaled tidal volume; FiO_2_, fraction of inspiration oxygen; SpO_2_, oxygen saturation; RCT, randomized controlled trial;PtcCO_2_, transcutaneous carbon dioxide tension; RSV, respiratory syncytial virus; HFNC, high-flow nasal cannula*.

Piastra et al. demonstrated the feasibility of using helmet NIPPV in children with hypoxemic ARF (53, 55). Helmet use improved oxygenation without major observable complications in four children and was well-tolerated in 13 immunocompromised children with acute respiratory distress syndrome (ARDS) (55).

A small prospective case series study, which enrolled eight infants with bronchiolitis, revealed that a helmet was suitable for delivering heliox with CPAP (54). Helmet CPAP can also be using as a post-extubation respiratory support in severely ill patients such as pediatric liver transplant patient (57). Moreover, a helmet can be used in patients with a contraindication to facemask use, such as those with a congenital facial deformity (58), or facial trauma (59), or who are undergoing otorhinolaryngological surgery (60).

### Exhaled Air Dispersion Distance

Health care workers are at high risk of contracting COVID-19 when performing daily respiratory care during the COVID-19 pandemic. Therefore, the ability to apply NIV using an interface with lower air leakage and a shorter air dispersion distance is essential (61–63). An *in vitro* experiment investigated two helmet interfaces with double tube limbs by examining the exhaled air dispersion distance, as marked by the smoke particles in an adult model. For the Sea-Long head tent (Sea-Long Medical Systems, TX, USA), the dispersion distance was 17–27 cm when the inspiratory positive airway pressure was set at 12–20 cmH_2_O; the CaStar-R StarMed (Intersurgical, Wokingham, UK) exhibited negligible air leakage (64). The study used smoke as the exhaled air marker; however, droplets are heavier, which may have led to overestimation (15, 64). *In vivo* studies have revealed that facemask NIV did not increase aerosol generation and dispersal compared with HFNC or conventional oxygen therapy (65, 66). Placement of nasogastric or orogastric tubes is a common measure for preventing aerophagia caused by NIV (27, 67–69). However, the presence of these tubes may increase air leakage and breaks in integrity when using NIV. The sealed access ports on the neck collar of the helmet can prevent air leakage (70); therefore, NIV delivered through a helmet may result in less air leakage and a shorter dispersion distance than conventional interfaces.

### Limitations of Helmet Ventilation

The large internal volume and high compliance of the polyvinyl chloride hood are common features of helmets; however, they may prevent the exhaled tidal volume from being reliably measured during helmet ventilation (32, 71, 72). Furthermore, claustrophobia may occur and be unavoidable during helmet ventilation; however, it has rarely been reported in the literature.

## Conclusion

The intention of the present article was not to suggest that helmets can replace conventional interfaces for NIV during the COVID-19 pandemic. Rather, this article supports the proposition that this interface can be more widely used as an alternative for non-invasive respiratory support, especially during the COVID-19 pandemic. Use of helmet ventilation has benefits, including higher tolerability, improved comfort, less air leakage. To improve the quality of respiratory care and provide a variety of NIV interfaces for pediatric patients, health care workers should learn how to use helmet ventilation and remain open to use of this unfamiliar treatment.

## Author Contributions

K-YC, Y-HC, and P-ZL designed the collection of the literature, coordinated, and supervised data extraction from each article. K-YC, Y-HC, and S-CM conceptualized and designed the study, drafted the initial manuscript, reviewed, and revised the manuscript. All authors approved the final manuscript as submitted and agreed to accountability for all aspects of the work.

## Funding

This work was supported by a research grant from Shin Kong Wu Ho-Su Memorial Hospital (2020SKHADR024). This funding source had no role in the design of this study and did not have any role during its execution, analyses, interpretation of the data, or decision to submit results.

## Conflict of Interest

The authors declare that the research was conducted in the absence of any commercial or financial relationships that could be construed as a potential conflict of interest.

## Publisher's Note

All claims expressed in this article are solely those of the authors and do not necessarily represent those of their affiliated organizations, or those of the publisher, the editors and the reviewers. Any product that may be evaluated in this article, or claim that may be made by its manufacturer, is not guaranteed or endorsed by the publisher.
