# Corrigendum: Helmet ventilation for pediatric patients during the COVID-19 pandemic: A narrative review

**DOI:** 10.3389/fped.2023.1168133

**Published:** 2023-03-20

**Authors:** Shu-Chi Mu, Yu-Hsuan Chien, Pin-Zhen Lai, Ke-Yun Chao

**Affiliations:** ^1^ Department of Pediatrics, Shin Kong Wu Ho-Su Memorial Hospital, Taipei, Taiwan; ^2^ School of Medicine, College of Medicine, Fu Jen Catholic University, New Taipei, Taiwan; ^3^ Department of Pediatrics, Fu Jen Catholic University Hospital, Fu Jen Catholic University, New Taipei, Taiwan; ^4^ Department of Respiratory Therapy, Fu Jen Catholic University Hospital, Fu Jen Catholic University, New Taipei, Taiwan; ^5^ School of Physical Therapy, Graduate Institute of Rehabilitation Sciences, Chang Gung University, Taoyuan, Taiwan

**Keywords:** helmet ventilation, pediatric, noninvasive respiratory support, continuous positive airway pressure, noninvasive positive pressure ventilation, coronavirus disease 2019, respiratory failure

A Corrigendum on Helmet ventilation for pediatric patients during the COVID-19 pandemic: A narrative review By Mu S-C, Chien Y-H, Lai P-Z and Chao K-Y (2022) Front. Pediatr. 10:839476. doi: 10.3389/fped.2022.839476

In the published article, there was a mistake in the “Exhaled Air Dispersion Distance” section. This sentence previously stated: “Placement of nasogastric or orogastric tubes is a common measure for preventing aerophobia caused by NIV (27, 67–69).”

The corrected sentence appears below: “Placement of nasogastric or orogastric tubes is a common measure for preventing aerophagia caused by NIV (27, 67–69).”

The authors apologize for this error and state that this does not change the scientific conclusions of the article in any way. The original article has been updated.

